# Case Report: Glossopharyngeal neuralgia secondary to syncope: insights into microvascular decompression treatment

**DOI:** 10.3389/fsurg.2026.1785296

**Published:** 2026-07-28

**Authors:** Ying Dang, Aichao Du, Jingjing Han, Zhe Shi

**Affiliations:** 1 The Second Hospital & Clinical Medical School, Lanzhou University, Lanzhou, Gansu, China; 2 Department of Neurosurgery, Second Hospital of Lanzhou University, Lanzhou, Gansu, China; 3 Lanzhou University, Lanzhou, Gansu, China

**Keywords:** glossopharyngeal neuralgia, glossopharyngeal-vagal nerve syndrome, microvascular decompression, neurovascular compression, syncope

## Abstract

This article reports a case of a 56-year-old male patient with glossopharyngeal-vagal nerve syndrome. The main clinical manifestations were paroxysmal, sharp, knife-like pain in the right root of the tongue and pharynx, which occurred synchronously with syncope without any premonitory dizziness or nausea during pain episodes. Preoperative imaging suggested neurovascular compression, with the right posterior inferior cerebellar artery traversing and compressing the rootlets of both the glossopharyngeal nerve and proximal vagus nerve. The patient underwent a right retrosigmoid craniotomy for microvascular decompression targeting both nerves. Postoperatively, the patient's tongue root pain and pain-induced syncope resolved completely. The patient recovered well without serious complications. Notably, 24-h Holter monitoring captured real-time synchronous sinus bradycardia strictly matched with pain attacks, providing direct objective neurophysiological evidence for the pain-triggered cardioinhibitory reflex. A 6-month long-term follow-up further confirmed sustained resolution of all symptoms, without sensory deficits, recurrent neuralgia or syncope. This case confirms that microvascular decompression is a safe and effective treatment for glossopharyngeal-vagal nerve syndrome, addressing the root cause of both pain and syncope. It also highlights a easily misdiagnosed clinical phenotype and provides novel electrophysiological and surgical references for this rare cranial nerve disorder.

## Introduction

Glossopharyngeal-vagal nerve syndrome is a rare cranial nerve disorder characterized by paroxysmal, severe pain in the glossopharyngeal region, which can be triggered by swallowing, talking, or coughing. Epidemiologically, this disease accounts for only 0.2%–1.3% of all facial and cranial neuralgias, representing an extremely rare posterior fossa neurovascular compression disorder compared with trigeminal neuralgia, which is caused by neurovascular conflict in 70%–90% of cases. A unique feature of this condition is that some patients may experience bradycardia, hypotension, or even syncope during pain episodes. This occurs because the pain stimulus reflexively induces cardiac inhibition via the vagus nerve, posing a potential life-threatening risk.

Similar to trigeminal neuralgia, the primary etiology in most cases is compression of the nerve root at its entry zone into the brainstem by a vascular loop (1). Although the two diseases share identical neurovascular compression pathogenesis and unified microvascular decompression (MVD) surgical strategies, glossopharyngeal-vagal neuralgia carries higher clinical risks due to the involvement of vagal-mediated autonomic reflexes that induce malignant cardioinhibitory syncope. Although carbamazepine may serve as an initial treatment option, microvascular decompression has become the preferred surgical intervention for patients with medication-refractory symptoms or those accompanied by syncope, as it addresses the underlying etiology and can achieve long-term relief or even a cure.

Most existing literature describes pain and syncope as sequential attacks in this disease (2), while cases presenting synchronous pain and syncope without pre-syncopal aura are rarely systematically discussed and easily misdiagnosed as simple vasovagal syncope. In addition, intraoperative dual compression of glossopharyngeal and vagus nerves, as well as objective electrophysiological evidence synchronizing pain and cardiac abnormalities, are seldom reported in detail. This paper reports a typical case of a patient who was successfully treated with microvascular decompression in the Department of Neurosurgery at Lanzhou University Second Hospital, with supplemented standardized surgical details, real-time synchronous electrophysiological evidence, analysis of diagnostic pitfalls, single-center surgical efficacy data and long-term follow-up outcomes to enrich the clinical and mechanistic evidence of this rare disease.

## Case presentation

A 56-year-old male was admitted to the hospital with the chief complaints of “paroxysmal severe pain in the right tongue base and pharynx for 2 years, accompanied by syncope for 3 months”. Two years prior, without an obvious precipitating cause, the patient began experiencing paroxysmal, electric shock-like, lancinating pain in the right tongue base, tonsillar fossa, and deep pharynx. Each episode lasted from several seconds to tens of seconds and could be triggered by swallowing, yawning, or speaking loudly. The initial frequency of attacks was low, and no systematic diagnosis or treatment was pursued. During the 2-year disease course, no identifiable lifestyle changes, newly administered medications, or acute intercurrent illnesses were observed, indicating that the symptomatic exacerbation was a spontaneous progression of chronic neurovascular compression and progressive cranial nerve hypersensitization.

In the recent three months, the frequency and intensity of the pain attacks increased significantly. Notably, approximately 50% of pain episodes were immediately followed by visual dimming and loss of consciousness, with no prodromal dizziness, nausea or fatigue before syncope. This synchronous onset pattern is a distinctive clinical feature of this case and a common diagnostic pitfall in clinical practice. The syncopal episodes lasted about 10 s–20 s, after which he regained consciousness spontaneously, without urinary or fecal incontinence or limb convulsions. One syncope episode resulted in a fall, causing soft tissue contusions to his head and face. Treatment with oral carbamazepine (up to a maximum dose of 600 mg/day) was ineffective in controlling the pain and led to adverse effects such as dizziness and drowsiness. Consequently, the patient sought surgical intervention.

Admission physical examination revealed stable vital signs, with no definitive positive findings on neurological assessment. Cardiovascular, pulmonary, and abdominal examinations were unremarkable. Otolaryngology evaluation identified no structural abnormalities in the pharyngolaryngeal region. Light mechanical stimulation of the right tonsillar fossa with a cotton swab elicited characteristic pain.

**Figure 1:**
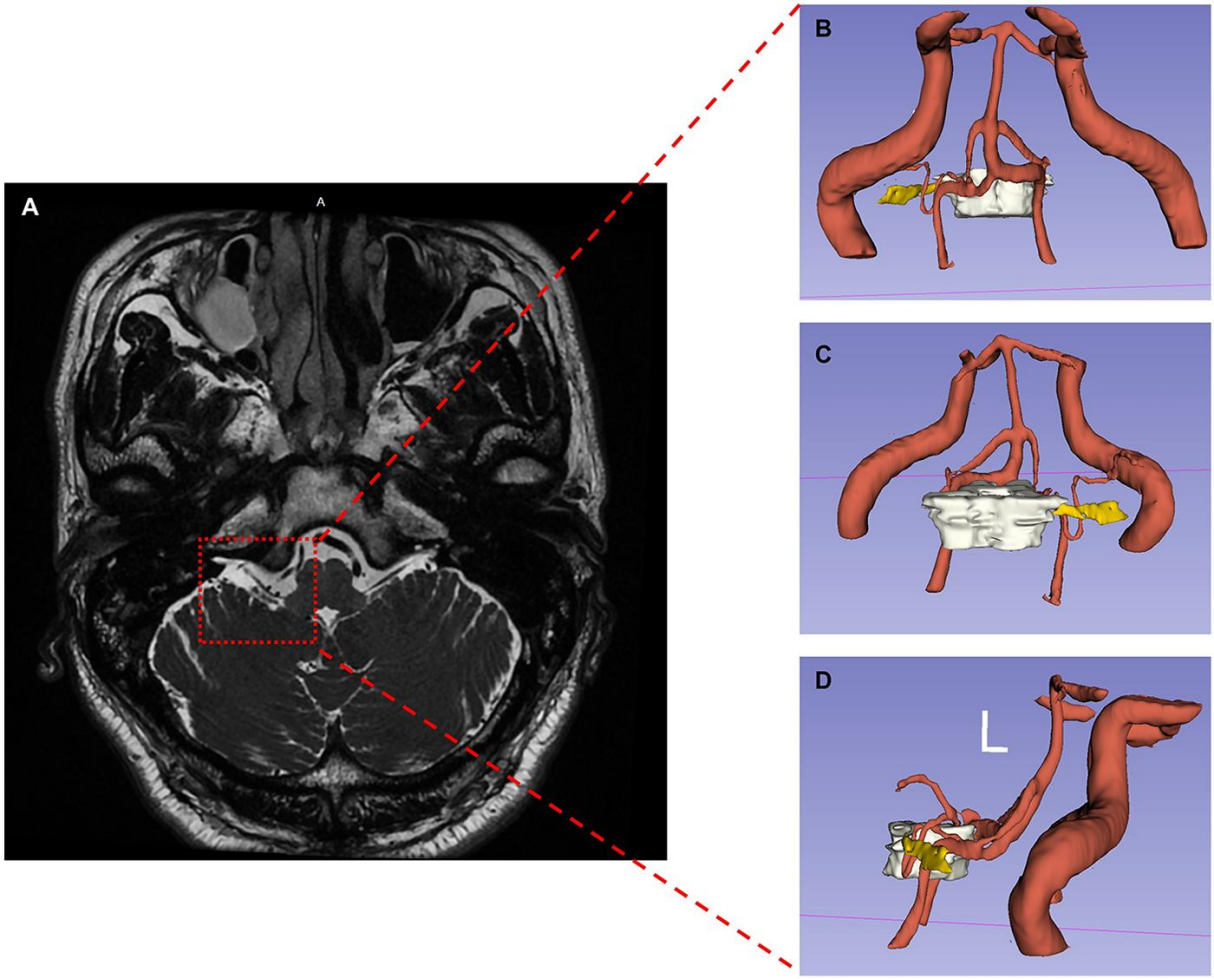
Magnetic resonance imaging and image post-processing of cranial nerves. All imaging images are acquired from the single patient in this case report. Arrow markers are added to explicitly label the precise neurovascular compression site at the right glossopharyngeal-vagal nerve rootlets. **(A)** Brain parenchyma MRI plain scan and cerebral MRA reveal no significant abnormalities. Additionally, bilateral maxillary sinusitis and ethmoid sinusitis are noted, along with a submucosal cyst in the right maxillary sinus. A neurovascular contact sign is observed at the root of the right glossopharyngeal nerve. **(B)** Anterior view from imaging post-processing, visualizing the contact between the glossopharyngeal nerve root and a small vascular structure. **(C)** Posterior view from imaging post-processing, demonstrating the neurovascular contact at the glossopharyngeal nerve root. **(D)** Right lateral view from imaging post-processing, further illustrating the neurovascular contact sign at the glossopharyngeal nerve root.

Cranial MRI/3D-TOF-MRA demonstrated vascular cross-compression at the root entry zone of both the glossopharyngeal and vagus nerves into the brainstem, caused by a loop of the right posterior inferior cerebellar artery (Figure 1). Intraoperative microscopic observation further confirmed the vascular indentation and nerve compression consistent with preoperative imaging findings. Conventional MRI and 3D-TOF-MRA sufficiently delineated the neurovascular conflict, and DTI or CISS sequences were not additionally performed due to clear imaging demonstration.

**Figure 2:**
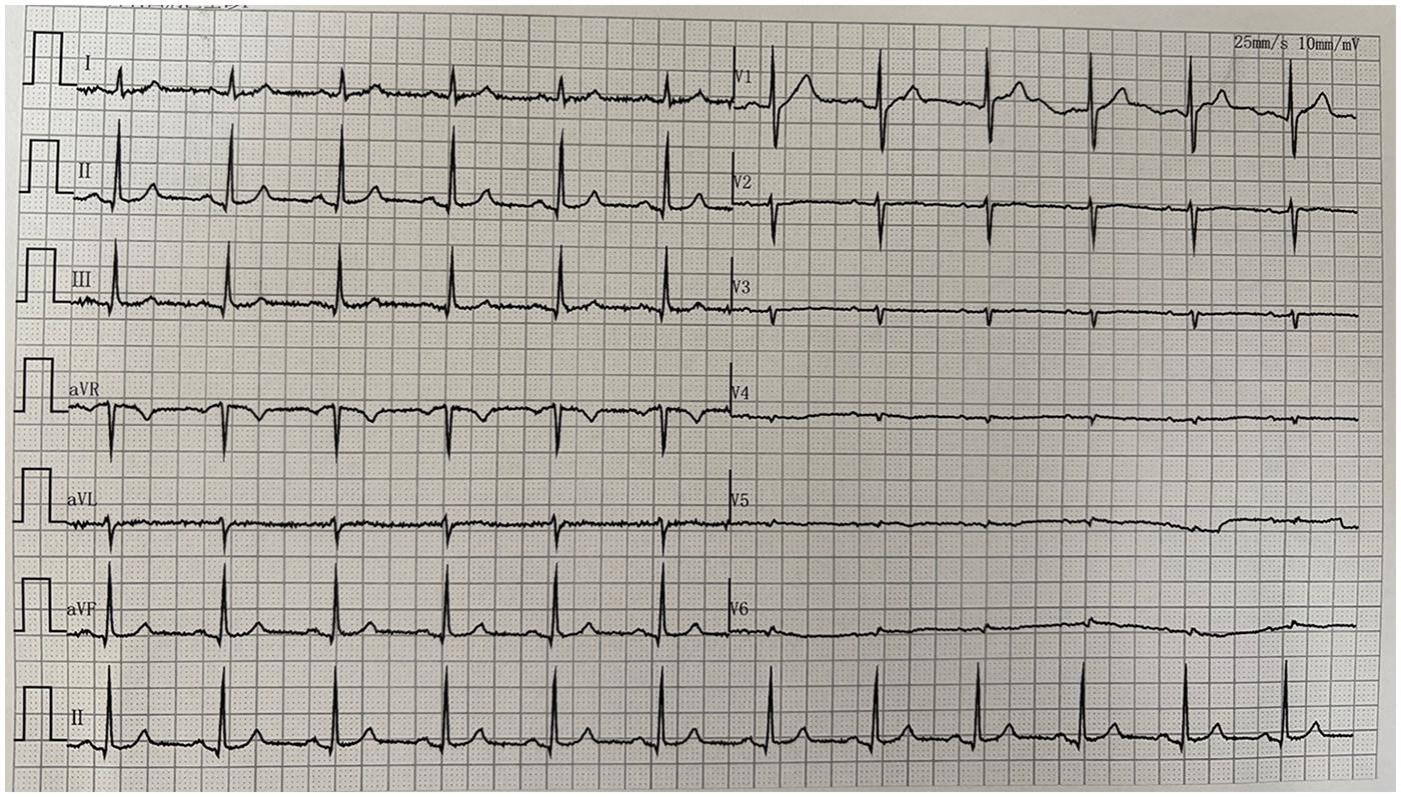
Electrocardiogram report. All ECG data are derived from the enrolled patient, showing normal baseline cardiac electrical activity, consistent with the patient's pain-free interval cardiac status. Heart rate: 71 (60–100) bpm; P-R interval: 183 (120–200) ms; SV1: 0.73 (normative range: 2–1) mV; RV5: 0.06 (0–2.5) mV; RV5 + SV1: 0.79 (0–4) mV; QRS duration: 90 (60–120) ms; QT/QTc: 384 (320–440)/418 (320–450) ms; Electrical axis: 72 (–30 to 90)°.

**Figure 3:**
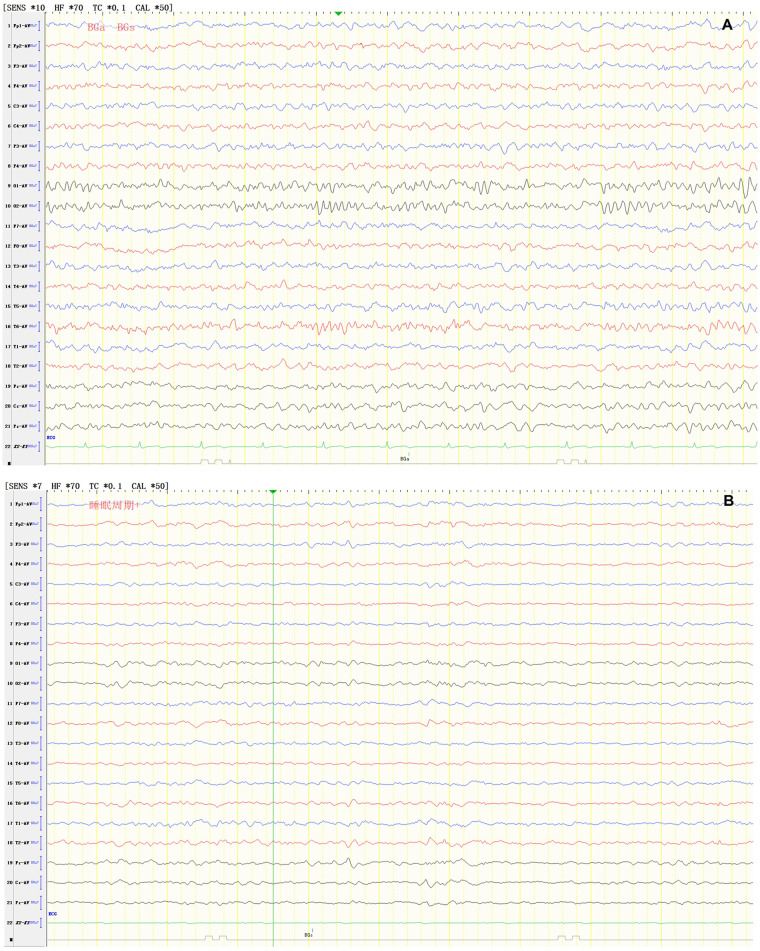
Normal electroencephalogram (EEG) results. All EEG examinations are performed on the current patient, excluding epileptogenic syncope etiology. **(A)** Posterior head dominant rhythm: 9–11 Hz alpha rhythm; Modulation/Amplitude: Modulation; acceptable low-to-moderate amplitude; bilateral symmetry; eye-opening suppression: Complete. **(B)** Recorded sleep stages. Sleep cycles: Generally normal. Sleep stages monitored: Stage I, Stage II, Stage III, REM stage.

Electrocardiogram (Figure 2) and 24-h Holter monitoring showed no significant baseline abnormalities. Crucially, 24-h Holter monitoring was synchronized with clinical attacks, and real-time sinus bradycardia was directly captured during each painful episode. This is objective *in vivo* evidence proving the causal link between pharyngeal pain and cardioinhibitory reflex. Electroencephalography was normal, excluding epileptogenic syncope (Figure 3). A diagnosis of glossopharyngeal-vagal neuralgia syndrome was established. Evaluation by the neurosurgical team confirmed clear indications for operative intervention.

The patient subsequently underwent a “right retrosigmoid craniotomy for exploration and microvascular decompression of the glossopharyngeal nerve and upper rootlets of the vagus nerve” under general anesthesia. Intraoperative neurophysiological monitoring, including lower cranial nerve electromyography and auditory brainstem response (ABR) monitoring, was routinely performed throughout the operation to prevent cranial nerve and brainstem injury. Intraoperative microscopic exploration revealed that a loop of the right posterior inferior cerebellar artery was tightly compressing both the glossopharyngeal nerve and the upper rootlets of the vagus nerve, with visible indentation and pallor at the site of neurovascular conflict. The offending PICA vascular loop was gently transposed and fully separated from the cranial nerve rootlets. A Teflon felt pledget was interposed between the vessel and nerves and fixed with a small amount of fibrin glue to avoid postoperative displacement, achieving adequate decompression.

Upon emergence from anesthesia, the patient reported immediate resolution of right-sided tongue base pain. The postoperative course was uneventful, with no complications such as hoarseness, aspiration, or dysphagia suggestive of lower cranial nerve injury. Throughout the first postoperative week, no further episodes of pain or syncope occurred, and the patient's general condition remained excellent. At the one-month telephone follow-up, the patient reported a complete return to normal daily activities, with no recurrence of pain or syncopal events. Further 6-month combined outpatient and telephone follow-up was completed. The patient maintained complete resolution of all symptoms, without any recurrent pain, syncope, or postoperative sensory disturbances including oropharyngeal numbness, foreign body sensation, taste abnormalities, or other neurological deficits.

## Discussion

Syncope resulting from glossopharyngeal-vagal nerve syndrome represents a distinct type of neurogenic syncope. Its mechanism involves the afferent transmission of pain impulses, which activate the dorsal motor nucleus of the vagus via the nucleus of the solitary tract, triggering a potent vagal reflex. This leads to a precipitous drop in heart rate and blood pressure, causing global cerebral hypoperfusion and subsequent syncope (3). According to the Polyvagal Theory, the mammalian social engagement system is governed by the ventral vagal complex. When this system is inhibited under stress, the organism sequentially resorts to more phylogenetically ancient systems—sympathetic mobilization and, ultimately, the dorsal vagal complex, which can precipitate syncope. The emergence of syncope signifies a regression of the neural regulatory hierarchy to a life-threatening, dysfunctional state, carrying a high risk of accidental injury or even sudden death. Therefore, active intervention is imperative (4, 5).

The diagnostic and therapeutic process in this case underscores the importance of precise diagnosis. The synchronous occurrence of pain and syncope without pre-syncopal aura is easy to be misdiagnosed as primary vasovagal syncope, which delays targeted neurosurgical treatment. Detailed patient history and the cotton swab trigger test are crucial for differential diagnosis. Imaging examinations, particularly high-resolution MRI, are essential for confirming neurovascular compression and guiding surgical planning. For patients with medication-refractory symptoms or those accompanied by syncope, microvascular decompression (MVD) is the preferred and most effective treatment (2, 6). This procedure aims to address the root cause by relieving the vascular compression rather than merely ablating the nerve. Consequently, it simultaneously eliminates the pain and abolishes the abnormal neural reflex responsible for syncope, while maximizing the preservation of neurological function.

Our single-center clinical data confirms the reliability of MVD for cranial neurovascular compression syndromes. Our neurosurgical center performs more than 300 MVD procedures annually for trigeminal neuralgia, hemifacial spasm, and glossopharyngeal neuralgia. For glossopharyngeal neuralgia with syncope, the immediate postoperative symptom relief rate exceeds 95%, the minor complication rate is less than 3% (mainly transient oropharyngeal numbness), and the long-term recurrence rate within 1–5 years is lower than 2%. Although the surgical site is adjacent to critical lower cranial nerves and the brainstem, the procedure was performed with the highest level of safety by the experienced neurosurgeons at Lanzhou University Second Hospital. The absence of any postoperative neurological deficits and the excellent outcome in this case fully demonstrate the superiority of this technique.

### Analysis of the mechanism of syncope related to glossopharyngeal neuralgia

Substantial literature and case reports consistently indicate that syncope triggered by glossopharyngeal neuralgia is fundamentally a form of reflex syncope characterized by a combination of both cardioinhibition and vasodepression, with a typically dominant and pronounced cardioinhibitory component (7). The core pathophysiological pathway leading to syncope begins with the noxious pain stimulus in the glossopharyngeal region, which is afferented by the glossopharyngeal nerve (8). A critical point is that after these afferent fibers enter the brainstem, they primarily project to the caudal portion of the nucleus of the solitary tract (NTS). This nucleus serves not only as a relay station for visceral sensation but also has close anatomical connections and functional integration with the adjacent dorsal motor nucleus of the vagus (DMNV) and the cardiovascular center.

Notably, it is essential to distinguish physiological and pathological Bezold–Jarisch (B-J) reflexes here. The physiological B-J reflex acts as a protective autonomic regulatory mechanism to maintain cardiovascular homeostasis under normal conditions. However, in glossopharyngeal-vagal neuralgia, chronic neurovascular compression induces persistent cranial nerve irritation, leading to maladaptive, excessive overactivation of the B-J reflex, which transforms the protective physiological response into a pathological life-threatening reflex that causes severe cardioinhibition and syncope (3, 9). When abnormally intense pain signals “flood” the NTS, they can erroneously lead to excessive activation of the DMNV—which is primarily responsible for cardioinhibition—through mechanisms of central convergence and facilitation (4). The activated DMNV subsequently generates excessive efferent impulses. These travel via the vagus nerve and its cardiac branches to innervate the heart, acting directly on the sinoatrial (SA) and atrioventricular (AV) nodes. This results in severe sinus bradycardia, sinus arrest, or high-grade atrioventricular block (10). The synchronous Holter data in this case directly verifies this pathological reflex pathway.

To avoid content deviation from the research theme, we simplify and focus only on core differential points closely related to this neurogenic syncope. Conventional vasovagal syncope is induced by emotion, postural change or general physical fatigue, while syncope in our patient is strictly triggered by localized pharyngeal pain; the cardioinhibitory response caused by glossopharyngeal-vagal neuralgia is more severe and persistent, and the two conditions have completely different etiologies and intervention strategies. Unlike primary cardiac syncope, which stems from organic heart diseases or primary cardiac electrophysiological disorders and presents abnormal baseline cardiac examinations, our patient had normal resting ECG and Holter results, with bradycardia occurring only synchronously with pain attacks. This confirms that the syncope was secondary to cranial nerve reflex rather than intrinsic cardiac lesions.

The pathophysiology of vasovagal syncope involves both cardioinhibition and vasodepression. Vasodepression arises from the reflexive inhibition of sympathetic outflow, leading to a sudden loss of peripheral vascular tone (11). Therefore, from a mechanistic standpoint, typical vasovagal syncope is not purely a “vasodepressor” or “cardioinhibitory” disorder, nor is it a form of structural heart disease. Instead, it constitutes a mixed response—triggered by a specific neural reflex—that incorporates both cardiac inhibition and vasodilation. This also explains why cardiac pacing alone is often insufficient to prevent syncope recurrence in many patients, as pacing can only correct bradycardia-induced syncope but cannot improve vagal-mediated vasodilation and subsequent hypotension, leaving patients at risk of persistent dizziness or presyncope.

### Typical vasovagal syncope

Vasovagal syncope is primarily triggered by factors such as fear, pain, or prolonged standing. Its hemodynamic profile can be vasodepressor, cardioinhibitory, or mixed. In young patients without significant heart disease, the mixed and vasodepressor types are more common; the degree of cardiac inhibition is typically relatively mild in these cases, though this is not an absolute rule (12). A core clinical distinction should be emphasized: conventional vasovagal syncope is induced by multimodal autonomic stress including emotional fluctuation, postural change, and physical fatigue, while glossopharyngeal-vagal neuralgia syncope is characterized by fixed, predictable, single pain-triggered reflex activation with far more severe and persistent cardioinhibition. Vasovagal syncope is triggered by emotional or orthostatic stress, whereas glossopharyngeal neuralgia syncope is triggered by specific craniofacial pain, involving a more distinct neural pathway. A key distinction lies in the intensity of cardiac inhibition, which is often extremely pronounced in glossopharyngeal neuralgia syncope (4). Electrocardiographic monitoring or implantable loop recorders frequently document prolonged asystole lasting several seconds or even over ten seconds, which is the direct “culprit” behind the syncopal episodes. In contrast, the decrease in heart rate is generally less severe in typical vasovagal syncope (13).

### Mechanism determines therapeutic strategy

The selection and efficacy of different treatments for glossopharyngeal neuralgia syncope further validate its underlying pathogenesis. Oral carbamazepine, by reducing aberrant pain signal transmission, can block the reflex arc at its source and is effective in some patients. For patients predominantly exhibiting cardiac inhibition, implanting a permanent cardiac pacemaker can effectively prevent syncope due to bradycardia. However, this approach does not address the pain itself or the vasodepressor component; patients may still experience dizziness or near-faint (presyncope) from persistent hypotension, which can be viewed as a manifestation of a “pacemaker syndrome”.

In contrast, microvascular decompression eliminates the source of abnormal impulses by relieving the neurovascular compression, thereby simultaneously curing the pain, cardiac inhibition, and vasodepression. Its superior efficacy serves as key evidence supporting the “neurogenic” nature of this syndrome (14). In the broader treatment of neurocardiogenic syncope, dual-chamber pacing with rate-drop response can significantly reduce syncope risk but has limited effect on presyncope; therefore, pacemaker therapy still requires cautious application.

Pharmacologically, beta-blockers, fludrocortisone, and alpha-agonists may be effective, supplemented by lifestyle modifications and physical maneuvers during prodromal symptoms. However, conducting large-scale controlled trials is challenging due to the spontaneous high variability in symptom frequency and unpredictable remission periods, creating a tension with the urgency of clinical decision-making. To address this, Santini et al. proposed a novel integrated device concept combining pacing and automated drug delivery (15). The ideal system should incorporate modules for diagnosis, patient-triggered intervention, automated drug delivery, dual-chamber pacing, and data storage. For instance, atropine is effective for cardioinhibitory syncope and shows a non-significant beneficial trend for vasodepressor types. However, given the diversity and dynamic nature of syncope types, even a “pharmacological pacemaker” would need to be equipped with multiple drugs to manage different events.

To further enrich the mechanistic and clinical framework of this study, we supplemented recent high-quality literature. Relevant evidence regarding cranial neurodynamics, vagal autonomic modulation, and multidisciplinary management of neurovascular compression syndromes is integrated to elaborate the biomechanical basis of cranial nerve irritation and the regulatory mechanism of the vagal-autonomic axis, strengthening the academic depth and clinical reference value of this case report (16).

## Conclusion

This literature review confirms that syncope associated with glossopharyngeal-vagal nerve syndrome represents a distinct clinical entity with a unique mechanism. It is not a traditional vasovagal or primary cardiac syncope, but should be precisely defined as a neurocardiogenic syncope triggered by specific cranial neuralgia and dominated by profound cardiac inhibition. The core pathophysiology involves the transmission of pain signals via the glossopharyngeal nerve, which abnormally activates the cardiac branches of the vagus nerve at the brainstem level, leading to severe bradycardia or sinus arrest, potentially coupled with vasodilation due to sympathetic inhibition.

Clarifying this mechanism is crucial: diagnostically, it necessitates establishing a clear temporal relationship between pain onset and syncope; therapeutically, it underscores that vascular compression of the nerve root is the initiating factor. Consequently, for patients presenting with syncope, microvascular decompression emerges as the preferred and ideal treatment, as it directly addresses the root cause, simultaneously eradicating both the pain and syncope. A profound understanding of this neuro-cardiac reflex pathway is key to avoiding misdiagnosis, formulating correct management strategies, and ultimately improving patient outcomes.

Collectively, this case report innovatively clarifies the pathological maladaptive B-J reflex mechanism, provides synchronized electrophysiological evidence for pain-triggered syncope, supplements single-center surgical efficacy data and long-term follow-up outcomes, and distinguishes the clinical differences between common neurovascular compression syndromes, offering valuable clinical references for the diagnosis and surgical treatment of rare glossopharyngeal-vagal nerve syndrome.

## Data Availability

The raw data supporting the conclusions of this article will be made available by the authors, without undue reservation.
